# Posttranslational Regulation of Human DNA Polymerase ι*

**DOI:** 10.1074/jbc.M115.675769

**Published:** 2015-09-14

**Authors:** Justyna McIntyre, Mary P. McLenigan, Ekaterina G. Frank, Xiaoxia Dai, Wei Yang, Yinsheng Wang, Roger Woodgate

**Affiliations:** From the ‡ Laboratory of Genomic Integrity, NICHD, National Institutes of Health, Bethesda, Maryland 20892-3371,; § Institute of Biochemistry and Biophysics, Polish Academy of Sciences, 02-106 Warsaw, Poland,; ¶ Department of Chemistry, University of California, Riverside, California 92521-0403, and; ‖ Laboratory of Molecular Biology, NIDDK, National Institutes of Health, Bethesda, Maryland 20892

**Keywords:** DNA repair, DNA synthesis, mutagenesis, posttranslational modification (PTM), ubiquitin, DNA polymerase ι, Y-family DNA polymerase

## Abstract

Human DNA polymerases (pols) η and ι are Y-family DNA polymerase paralogs that facilitate translesion synthesis past damaged DNA. Both polη and polι can be monoubiquitinated *in vivo*. Polη has been shown to be ubiquitinated at one primary site. When this site is unavailable, three nearby lysines may become ubiquitinated. In contrast, mass spectrometry analysis of monoubiquitinated polι revealed that it is ubiquitinated at over 27 unique sites. Many of these sites are localized in different functional domains of the protein, including the catalytic polymerase domain, the proliferating cell nuclear antigen-interacting region, the Rev1-interacting region, and its ubiquitin binding motifs UBM1 and UBM2. Polι monoubiquitination remains unchanged after cells are exposed to DNA-damaging agents such as UV light (generating UV photoproducts), ethyl methanesulfonate (generating alkylation damage), mitomycin C (generating interstrand cross-links), or potassium bromate (generating direct oxidative DNA damage). However, when exposed to naphthoquinones, such as menadione and plumbagin, which cause indirect oxidative damage through mitochondrial dysfunction, polι becomes transiently polyubiquitinated via Lys^11^- and Lys^48^-linked chains of ubiquitin and subsequently targeted for degradation. Polyubiquitination does not occur as a direct result of the perturbation of the redox cycle as no polyubiquitination was observed after treatment with rotenone or antimycin A, which both inhibit mitochondrial electron transport. Interestingly, polyubiquitination was observed after the inhibition of the lysine acetyltransferase KATB3/p300. We hypothesize that the formation of polyubiquitination chains attached to polι occurs via the interplay between lysine acetylation and ubiquitination of ubiquitin itself at Lys^11^ and Lys^48^ rather than oxidative damage *per se*.

## Teaser

**Background:** Many proteins are subject to posttranslational regulation, such as ubiquitination.

**Results:** Human DNA polymerase ι (polι) can be monoubiquitinated at >27 unique sites, and exposure to naphthoquinones results in polyubiquitination of polι.

**Conclusion:** Ubiquitination sites are located across the entire polι polypeptide as well as various structural motifs.

**Significance:** Ubiquitination at these sites is likely to alter cellular functions of polι *in vivo*.

## Introduction

To survive the constant threat to their genomes from exposure to endogenous and exogenous DNA-damaging agents, cells are equipped with an impressive array of DNA repair mechanisms. However, situations arise where DNA lesions in the genome remain unrepaired and cells are forced to tolerate the DNA damage. One such tolerance mechanism is “translesion DNA synthesis” (TLS).^2^ During TLS, the high fidelity replicase, which is unable to traverse the DNA lesion due to its constrained active site, is replaced with a specialized DNA polymerase (pol) with a more spacious active site that can accommodate the damaged DNA (1). Many of the DNA polymerases discovered in the past 15 years appear to have some capacity to promote TLS. However, the best characterized TLS polymerases belong to the Y-family of DNA polymerases (2). Y-family DNA polymerases are typified by human polη, which bypasses a thymine-thymine cyclobutane pyrimidine dimer efficiently and with much higher accuracy than any other human TLS polymerases (3). Because of their more spacious active sites (4), the TLS enzymes are also able to accommodate non-canonical Watson-Crick base pairing and are usually much more error-prone than high fidelity replicases when they replicate undamaged DNA (1). In specialized situations, such as during immunoglobulin somatic hypermutation, this creates genetic diversity and leads to high affinity antigen-specific immunoglobulins (5). However, under normal circumstances, random mutagenesis of chromosomal DNA is highly deleterious, often leading to mutagenesis and tumorigenesis in higher organisms.

It is clear, therefore, that the activity of the TLS polymerases needs to be tightly regulated so that they only gain access to undamaged genomic DNA when appropriate. Previous studies have revealed that the posttranslational modification of the TLS polymerases themselves or their interacting partners plays a major role in regulating their cellular activities (for a review, see Ref. 6). In particular, both mono- and polyubiquitination appear to play a central role in regulating TLS polymerases because monoubiquitination of the proliferating cell nuclear antigen (PCNA) appears to control the switch between high fidelity replicases and TLS polymerases (7, 8). However, PCNA can be further polyubiquitinated. The addition of Lys^63^-linked polyubiquitin chains to PCNA that is monoubiquitinated at Lys^164^ leads to a damage avoidance template-switching pathway that in contrast to TLS allows for error-free DNA damage bypass (9, 10).

In addition to PCNA, both human polη and polι TLS polymerases are also subject to monoubiquitination (11, 12). Attaching a single ubiquitin moiety to one of four lysine residues in the C terminus of polη blocks the physical interaction between polη and PCNA (12). As a consequence, polη needs to be actively deubiquitinated prior to interacting with PCNA and subsequently recruited to a stalled replication fork (12). The cellular role of polι monoubiquitination remains enigmatic. However, our previous results suggest that monoubiquitination of either polη or polι is a prerequisite for the physical and functional interaction between the two polymerases (13).

Human polι is one of the least accurate DNA polymerases and exhibits a 10,000-fold range in base substitution fidelity depending on the template sequence copied (for a review, see Ref. 1). Polι has been extensively characterized at the biochemical level (14–19), and its *in vivo* relocalization in response to DNA damage has been shown (20, 21). The enzyme is involved in the error-free bypass of methylglyoxal-induced minor groove lesions, *N*^2^-carboxyethyl-2′-deoxyguanosine (22), and a deficiency in polι has been suggested to cause sensitivity to oxidative and 4-hydroxynonenal DNA damage (23, 24) as well as stimulate UV-induced mesenchymal carcinogenesis (25). However, the primary biological function of polι is still far from being understood.

Some assumptions on the cellular role of polι can be derived from the various domains/motifs identified in polι. The N-terminal part of the protein contains two partly overlapping catalytic domains, a DNA polymerase domain and a 5′-deoxyribose phosphate lyase domain (26, 27). The core polymerase domain is built of palm, finger, thumb, and little finger subdomains (28, 29). The C-terminal portion of the protein is unstructured and devoted to facilitating interactions with a variety of proteins. Similar to other TLS polymerases, polι contains a PCNA-interacting peptide motif responsible for the interaction with PCNA (30–32) and a Rev1-interacting region (33, 34). It also contains two ubiquitin binding motifs (11).

Typically, the conjugation of ubiquitin to the lysine residue of a substrate protein occurs as a result of a three-enzyme cascade process involving ubiquitin-activating enzyme (E1), ubiquitin-conjugating enzyme (E2), and ubiquitin ligase (E3) (35). Ubiquitin contains seven lysine residues (Lys^6^, Lys^11^, Lys^27^, Lys^29^, Lys^33^, Lys^48^, and Lys^63^) and is itself a target for further ubiquitination. Indeed, repetitive ubiquitination can establish polyubiquitin chains on a target protein. The length, type of linkage, and consequent shape of conjugated polyubiquitin chains direct the function and processing of many intracellular proteins in eukaryotes (for a review, see Ref. 36). All types of ubiquitin chains exist in the cell; however, they vary in abundance and functionality. Different types of polyubiquitin chains regulate different biological processes by promoting proteasomal degradation, altering subcellular localization, modulating enzymatic activity, and facilitating protein-protein interactions (37).

In the current work, we used mass spectrometry analysis to identify the lysine residues that can be ubiquitinated in human polι. In contrast to PCNA, which is primarily ubiquitinated at Lys^164^, and polη where the ubiquitinated residues are clustered in its C terminus, the monoubiquitinated residues in polι are scattered among its various functional domains/motifs. Furthermore, unlike monoubiquitinated polη that is deubiquitinated upon UV irradiation, the level of monoubiquitinated polι remains unchanged after exposure to UV irradiation, ethyl methanesulfonate, mitomycin C, or potassium bromate. Interestingly, however, after exposure to menadione and structurally related naphthoquinones, polι is rapidly polyubiquitinated, and intracellular levels of both the unmodified and the monoubiquitinated forms of polι decrease significantly. We present evidence that the polyubiquitination of polι is not in response to oxidative DNA damage *per se* but is rather due to the inhibition of KAT3B/p300-dependent acetylation of ubiquitin, which in turn allows for the formation of Lys^11^- and Lys^48^-linked polyubiquitin chains on polι that subsequently target it for degradation.

## Experimental Procedures

### 

#### 

##### Reagents

2,3-Dimethoxy-1,4-naphthoquinone (DMNQ) was purchased from Enzo Life Sciences, and ethyl methanesulfonate, mitomycin C, potassium bromate, menadione, 1,4-naphthoquinone, juglone, plumbagin, L002, rotenone, and antimycin A were all purchased from Sigma-Aldrich.

##### Mammalian Expression Plasmids

Plasmid pJRM46 is a derivative of pCMV6AN-DDK (Origene Technologies, Rockville, MD), which expresses N-terminal FLAG-tagged full-length human polι (13). Derivatives with single or multiple Lys → Ala or Lys → Arg substitutions were generated by chemically synthesizing appropriate DNA fragments (Genscript) that were subsequently cloned into pJRM46. Plasmid pRK7-POLI-3XFLAG is a derivative of pRK7 (38), which expresses full-length human polι with three C-terminal FLAG tags. The vector was constructed by inserting three tandem repeats of the FLAG epitope tag (DYKDDDDK) into the BamHI and EcoRI sites of pRK7 to generate pRK7–3XFLAG. The full-length human *POLI* gene was amplified from HEK293T cells by reverse transcription-PCR using primers POLI-S (AAAGCTAGCATGGAGAAGCTGGGGGTGGA) and POLI-AS (AAAGGATCCTTTATGTCCAATGTGGAAATCT). These primers introduce 5′ NheI and 3′ BamHI sites into the amplicon, which was subcloned into the XbaI and BamHI sites of the pRK7–3XFLAG vector. A full list of plasmids used in the current study is shown in Table 1.

**TABLE 1:** **Plasmids used in this study**

Plasmid	Description	Source/Ref.
pJRM46	pCMV6AN-DDK-polι (N-terminal tag)	13
pRK7-POLI-3XFLAG	pRK7–3XFLAG-polι (C-terminal tag)	This work
pJRM57	pCMV6AN-DDK-polι_K248A	This work
pJRM48	pCMV6AN-DDK-polι_K522A	This work
pJRM49	pCMV6AN-DDK-polι_K526A	This work
pJRM50	pCMV6AN-DDK-polι_K530A	This work
pJRM51	pCMV6AN-DDK-polι_K549A	This work
pJRM52	pCMV6AN-DDK-polι_K704A	This work
pJRM53	pCMV6AN-DDK-polι_K715A	This work
pJRM226	pCMV6AN-DDK-polι_K715R	This work
pJRM54	pCMV6AN-DDK-polι_K704A/K715A	This work
pJRM55	pCMV6AN-DDK-polι_K522A/K526A/K530A/K549A	This work
pJRM89	pCMV6AN-DDK-polι_K248A/K522A/K526A/K530A/K549A/K704A/K715A	This work
pJRM106	pCMV6AN-DDK-polι_K237A/K245A/K248A/K267A/K271A/K522A/K526A/K530A/K549A/K550A/K704A/K715A	This work
pJRM219	pCMV6AN-DDK-polι_K237R/K245R/K248R/K267R/K271R/K522R/K526R/K530R/K549R/K550R/K704R/K715R	This work
pJRM193	pCMV6AN-DDK-polι_K51A/K53A/K72A/K237A/K245A/K248A/K267A/K271A/K283A/K309A/K310A/K320A/K522A/K526A/K530A/K549A/K550A/K704A/K715A	This work
pJRM192	pCMV6AN-DDK-polι_K51R/K53R/K72R/K237R/K245R/K248R/K267R/K271R/K283R/K309R/K310R/K320R/K522R/K526R/K530R/K549R/K550R/K704R/K715R	This work

##### Plasmid Transfection, Protein Expression, and Western Blotting

HEK293T cells were plated onto 100-mm culture plates at a seeding density of 3 × 10^6^ cells. When cells were ∼40% confluent, plasmids were transfected into cells using Turbofectin 8.0 according to the manufacturer's instructions (Origene Technologies). Cells were either mock treated or exposed to a variety of agents 24 or 48 h after transfection depending upon the treatment times required. At appropriate times thereafter, cells were gently collected, washed twice with cold Dulbecco's PBS without calcium or magnesium, suspended in modified radioimmunoprecipitation assay buffer (RIPA buffer) (25 mm Tris-HCl, pH 7.6, 150 mm NaCl, 1% Nonidet P-40, 1 mm EDTA, 1 mm PMSF, 1 mm Na_3_VO_4_, and Sigma protease inhibitor mixture), and lysed by sonication for 10 s. Immediately after sonication, the extracts were clarified by centrifugation at +4 °C for 15 min in a Sorvall Biofuge Pico at 16,000 × *g*. The supernatants (extracts) were transferred to fresh tubes, and protein concentrations were measured using the Pierce BCA Assay (Pierce Biotechnology). Cell extracts were kept at +4 °C until being separated by 4–16% gradient SDS-PAGE. Proteins were transferred to a PVDF membrane, and FLAG-tagged polι was visualized using a Tropix Western-Star chemiluminescence kit using mouse anti-FLAG monoclonal antibodies (Abnova) followed by secondary anti-mouse antibody (Novagen). Where noted, the following other antibodies were used: POLI monoclonal antibody (M01) clone 8G9 (Abnova) or polyclonal rabbit antibodies raised against a keyhole limpet hemocyanin-conjugated peptide corresponding to the C-terminal 15 amino acid residues of polι (20). Where noted, the level of polι was compared with β-actin present in the extracts that was visualized using rabbit anti-β-actin antibodies (Cell Signaling Technology). The intensity of the individual bands was quantified using the ImageJ 1.47 application (National Institutes of Health).

##### Mass Spectrometry Analysis

**FIGURE 1.**
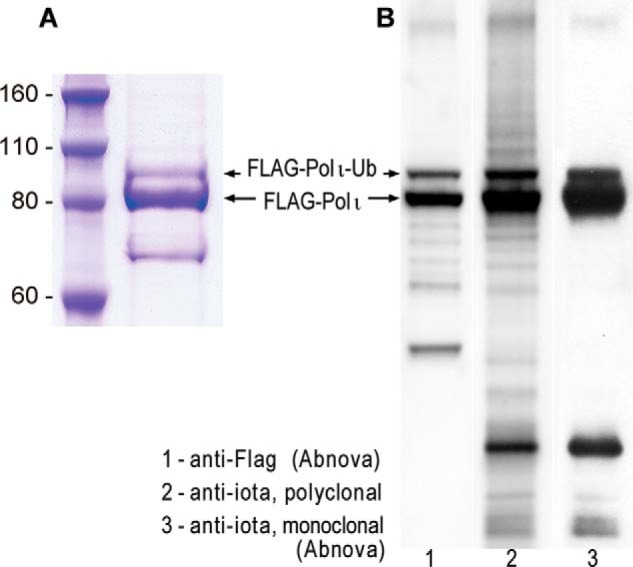
**Highly purified polι protein purchased from Origene Technologies.** *A*, Coomassie Brilliant Blue-stained gel of FLAG-tagged polι purified from HEK293T cells. *B*, Western blot of purified polι. Polι was visualized using monoclonal antibodies to the FLAG epitope (*lane 1*), polyclonal antibodies to polι (*lane 2*), or monoclonal antibodies to polι (*lane 3*). The images clearly show that the major band observed in the Coomassie Brilliant Blue-stained gel corresponds to native FLAG-polι. The slower migrating band also contains polι and appears to be a posttranslationally modified form of polι. The faster migrating band observed in *A* does not appear to be related to FLAG-polι because it does not cross-react to either the FLAG or polι antibodies. *Ub*, ubiquitin.

Purified recombinant N-terminal FLAG-tagged human polι was purchased from Origene Technologies and was supplied at a final concentration of 0.106 mg/ml. Roughly 80% of the total protein represents unmodified FLAG-polι, and ∼10% represents a slower migrating modified FLAG-polι (Fig. 1*A*). A total of 5 μg of the combined FLAG-polι preparation was applied to a 1-mm-thick precast 10% polyacrylamide NuPAGE gel (Life Technologies). Proteins were separated by SDS-PAGE by running the gel at 190 V for 3.75 h. All subsequent solutions were prepared using ultrapure HPLC water. The gel was lightly stained using the Novex Collodial Blue Staining kit (Life Technologies) in a brand new disposable plastic tray. Unmodified and modified FLAG-polι bands were excised using a brand new hard backed razor blade under water. Gel fragments were washed two times with 50% acetonitrile in ultrapure water. Samples were sent to the Harvard Microchemistry Department (Harvard University, Cambridge, MA) where they were analyzed by mass spectrometry as a custom contract service.

pRK7-POLI-3XFLAG (1.5 μg) was transfected into HEK293T cells (70–80% confluence in a 6-well plate) using Lipofectamine 2000 (Invitrogen). After a 48-h incubation, the C-terminal 3XFLAG-tagged polι protein was isolated and purified using anti-FLAG M2 beads (Sigma). The purified protein was digested with trypsin (Roche Applied Science) at an enzyme/substrate ratio of 1:50 and subjected to LC-MS/MS analysis.

LC-MS/MS experiments were performed as described previously (39). Briefly, the peptides were separated on an EASY-nLC II and analyzed on an LTQ Orbitrap Velos mass spectrometer equipped with a nanoelectrospray ionization source (Thermo). The trapping column (150 μm × 50 mm) and separation column (75 μm × 120 mm) were packed with ReproSil-Pur C_18_-AQ resin (3 μm in particle size; Dr. Maisch HPLC GmbH, Germany). The peptide samples were first loaded onto the trapping column in CH_3_CN/H_2_O (2:98, v/v) at a flow rate of 4.0 μl/min and resolved on the separation column with a 120-min linear gradient of 2–40% acetonitrile in 0.1% formic acid and at a flow rate of 300 nl/min. The LTQ-Orbitrap Velos mass spectrometer was operated in the positive ion mode, and the spray voltage was 1.8 kV. The full-scan mass spectra (*m*/*z* 300–2000) were acquired with a resolution of 60,000 at *m*/*z* 400 after accumulation to a target value of 500,000 in the linear ion trap. MS/MS data were obtained in a data-dependent scan mode where one full MS scan was followed with 20 MS/MS scans.

## Results

### 

#### 

##### Sites of Ubiquitination in Polι

It has been reported previously that polι is monoubiquitinated *in vivo* (11). However, at the time that we embarked on these studies, the location of the modified residue(s) had yet to be determined. To identify the site(s) of ubiquitination in polι, we initially utilized the contract services of the Harvard Microchemistry Department (Harvard University) to provide mass spectrometry analysis of a commercially available preparation of N-terminal FLAG-tagged human polι (Origene Technologies) (Fig. 1*A*). The preparation contained a significant amount of a slower migrating protein that cross-reacts with anti-FLAG antibodies as well as both polyclonal and monoclonal antibodies against polι (Fig. 1*B*). Based upon the earlier work of Bienko *et al.* (11), we hypothesized that the slower migrating band was likely to be monoubiquitinated polι.

Mass spectrometry analysis of the isolated slower migrating FLAG-polι protein indicated it was indeed monoubiquitinated polι, which was modified at six unique lysine residues (Lys^248^, Lys^522^, Lys^526^, Lys^530^, Lys^549^, and Lys^704^). Although we had limited mass spectrometry coverage of the very C terminus of polι, we rationalized that the C-terminal Lys^715^ residue might also be subject to ubiquitination because it is most probably localized on the surface of the protein and thus likely to be exposed to ubiquitinating enzymes.

**FIGURE 2.**
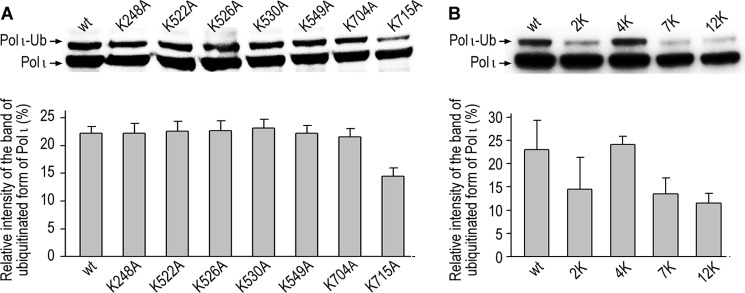
**Effects of Lys → Ala substitutions on the extent of polι ubiquitination in HEK293T cells.** *A*, single amino acid substitutions. *B*, multiple amino acid substitutions. *2K*, K704A/K715A; *4K*, K522A/K526A/K530A/K549A; *7K*, K248A/K522A/K526A/K530A/K549A/K704A/K715A; *12K*, K237A/K245A/K248A/K267A/K271A/K522A/K526A/K530A/K549A/K550A/K704A/K715A. *Upper panel*, a representative Western blot using monoclonal anti-FLAG antibodies. *Lower panel*, densitometric quantification of polι monoubiquitination. Data are the mean values from six (*A*) or three (*B*) independent experiments ±S.D. *Ub*, ubiquitin.

To determine which of the residues might be the primary site of polι ubiquitination, we transfected human HEK293T cells with a series of recombinant plasmids carrying FLAG-tagged polι, each containing a single lysine to alanine substitution (K248A, K522A, K526A, K530A, K549A, K704A, and K715A), and checked the extent of polι ubiquitination by Western blotting and probing with anti-FLAG antibodies. Remarkably, most of the polι mutants were ubiquitinated at levels comparable with the wild-type protein (Fig. 2*A*). Interestingly, the largest reduction in ubiquitination occurred in the K715A mutant, which exhibited ∼60% of the level observed with the wild-type protein, indicating that Lys^715^ is indeed a target for ubiquitination. Based on these observations, it appears that none of the seven lysines is an exclusive site of ubiquitination. However, we rationalized that modification at lysines in close proximity to the respective alanine substitution might mask the effect of individual lysine mutations (*e.g.* Lys^522^, Lys^526^, and Lys^530^). To test this hypothesis, we determined the extent of ubiquitination of polι mutants containing multiple Lys → Ala substitutions. This included double (K704A/K715A), quadruple (K522A/K526A/K530A/K549A), and even septuple (K248A/K522A/K526A/K530A/K549A/K704A/K715A) substitutions (Fig. 2*B*). To our surprise, ubiquitination of polι in the septuple mutant was only diminished by ∼50% compared with the wild-type protein, suggesting the existence of additional ubiquitination sites in polι. By analogy to polη where a K682A substitution leads to ubiquitination at nearby lysine residues (12), we made Lys → Ala substitutions at polι residues Lys^237^, Lys^245^, and Lys^550^. Based on the results obtained by Wagner *et al.* (40), who reported proteome-wide analysis of *in vivo* ubiquitination sites, we also made Lys → Ala substitutions at Lys^267^ and Lys^271^. However, a duodecuple mutant carrying all 12 Lys → Ala substitutions did not prevent monoubiquitination of polι (Fig. 2*B*), thereby implying additional sites of ubiquitination in polι.

We therefore undertook an independent mass spectrometry analysis approach, this time using C-terminal FLAG-tagged polι. Interestingly, we identified six ubiquitination sites in polι that were clustered in the N-terminal half of the polymerase (Lys^53^, Lys^283^, Lys^309^, Lys^271^, Lys^310^, and Lys^320^). None of these sites emerged in the original analysis of N-terminal FLAG-tagged polι performed at the Harvard Microchemistry Department, and only one residue (Lys^271^) was identified in the earlier studies by Wagner *et al.* (40).

**FIGURE 3.**
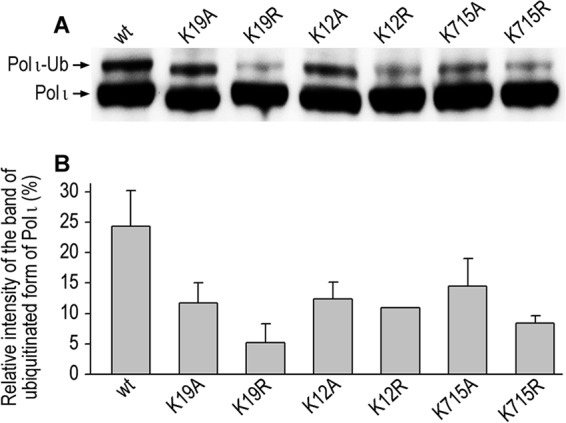
**Effects of Lys → Ala and Lys → Arg substitutions on the extent of ubiquitination of polι in HEK293T cells.** *A*, Western blot using monoclonal anti-FLAG antibodies. *B*, *lower panel*, densitometric quantification of polι monoubiquitination. Data are the mean values from four to five independent experiments ±S.D. Lysine residues were changed to either alanine orarginine: Lys^51^/Lys^53^/Lys^72^/Lys^237^/Lys^245^/Lys^248^/Lys^267^/Lys^271^/Lys^283^/Lys^309^/Lys^310^/Lys^320^/Lys^522^/Lys^526^/Lys^530^/Lys^549^/Lys^550^/Lys^704^/Lys^715^ (*K19*), Lys^237^/Lys^245^/Lys^248^/Lys^267^/Lys^271^/Lys^522^/Lys^526^/Lys^530^/Lys^549^/Lys^550^/Lys^704^/Lys^715^ (*K12*),and the C-terminal Lys^715^ residue. *Ub*, ubiquitin.

Thus, by three independent approaches, two specifically focused on polι (described herein) and one proteome-wide (Wagner *et al.* (40)), 17 independent ubiquitination sites in polι were identified. We were interested in determining whether substitutions at these sites would finally block ubiquitination of polι. Given the close structural proximity of polι Lys^51^ and Lys^72^ residues to Lys^53^ (29), we also made substitutions at these residues. The combined mutant has 19 Lys → Ala substitutions. We noted that this mutant has altered gel electrophoretic mobility (Fig. 3*A*) and decided to limit the influence of the multiple alanine substitutions on the global charge of the protein and consequently its structure, so we also generated a 19-Lys → Arg mutant.

Interestingly, the 19-Lys→ Ala mutant showed 60% higher levels of polι ubiquitination than the 19-Lys → Arg mutant (Fig. 3), suggesting that multiple Lys → Ala mutations probably changed the structure of polι and possibly exposed lysine residues that perhaps would not normally be subject to monoubiquitination. We also observed the same effect when comparing the 12-Lys → Ala with the 12-Lys → Arg mutants. In light of the fact that the Lys → Arg changes in the combined polι mutants reduced ubiquitination of polι more acutely than the Lys → Ala mutations, we decided to re-evaluate the effect of a single K715R substitution because the K715A mutant gave the greatest reduction in the levels of ubiquitination (Fig. 2). Again, the conservative K715R substitution diminished ubiquitination of polι to a greater extent than K715A. In some regards, this is surprising because one might expect that the extreme C-terminal residue would be exposed and there would not be a large structural effect of the alanine or arginine substitutions.

Recent technical progress in mass spectrometry-based methods in combination with novel ubiquitin enrichment strategies using di-Gly-Lys-specific antibody (41) have significantly increased the number of documented ubiquitinated proteins and pinpointed many of their ubiquitin-modified lysines on a global level, including many TLS proteins (6). Within the last 4 years, several groups have reported large scale detection of lysine ubiquitination events in human cells, and in nine of these studies, ubiquitination sites of polι have been identified (41–49).

**FIGURE 4.**
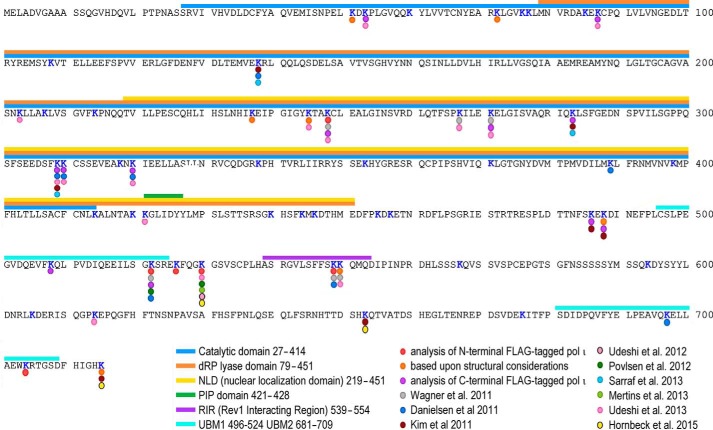
**Sites of ubiquitination in polι**. Lysine residues in polι that have been shown to be subject to ubiquitination are indicated with a *colored dot.* The reference for each residue is given below the primary amino acid sequence of polι. As noted, polι can be ubiquitinated at more than 27 unique lysine residues. Some residues have been observed in multiple studies, but no single residue has been identified in every study. The various motifs and domains in polι are identified by *color-coded bars* above the primary amino acid sequence. *dRP*, 5′-deoxyribose phosphate; *PIP,* PCNA-interacting peptide motif.

Fig. 4 summarizes all the lysine residues in polι that have been shown to be ubiquitinated. In total, 27 lysine residues of polι have been experimentally shown to be ubiquitinated. Based upon structural considerations, we have identified another three lysine residues that could potentially be ubiquitinated. Eight of the sites were detected just once. The remaining 19 ubiquitination sites in polι were identified in anywhere between two and six independent studies, often using very different experimental strategies (*e.g.* ectopically expressed *versus* chromosomally expressed polι and/or different detection methods). However, no single site has been identified in all of the posttranslational modification studies. Thus, unlike polη, which is ubiquitinated at one primary site and a handful of secondary sites (12), polι does not appear to possess a primary site for ubiquitination but is instead ubiquitinated at multiple lysine residues.

##### Polι Ubiquitination in Response to a Variety of DNA-damaging Agents

**FIGURE 5.**
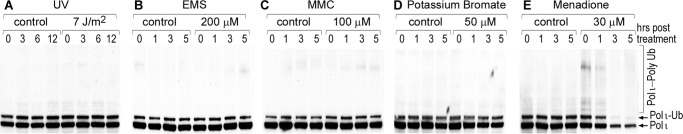
**Effect of DNA-damaging agents on the extent of polι ubiquitination in HEK293T cells.** *A*, UV irradiation (resulting in cyclobutane pyrimidine dimers and 6-4 photoproducts); *B*, ethyl methanesulfonate (*EMS*) (resulting in alkylation DNA damage); *C*, mitomycin C (*MMC*) (resulting in interstrand cross-links); *D*, potassium bromate (resulting in oxidative DNA damage); *E*, menadione (believed to cause oxidative damage and a variety of other cellular effects). Polι was visualized in Western blots using monoclonal antibodies to the N-terminal FLAG epitope. The major band is unmodified polι followed by monoubiquitinated polι. Slower migrating proteins are believed to be polyubiquitinated forms of polι. *Ub*, ubiquitin.

The 30 potential ubiquitination sites are distributed along the entire length of the polι peptide. However, some are clustered in domains and motifs of polι that are important for its cellular function in DNA damage tolerance (5′-deoxyribose phosphate lyase domain, catalytic polymerase domain, PCNA-interacting peptide motif, Rev1-interacting region, UBM1, and UBM2) (Fig. 4). We were therefore interested in determining whether the ubiquitination status of polι is influenced by exposure to DNA-damaging agents. Indeed, there is a precedent for damage-induced deubiquitination of human polη to allow it to interact with ubiquitinated PCNA and facilitate TLS (12). We therefore examined polι ubiquitination in response to treatment with agents that cause different types of DNA damage such as UV irradiation, which results in both cyclobutane pyrimidine dimers and 6-4 photoproducts; ethyl methanesulfonate, which generates alkylation damage (50); mitomycin C, which generates interstrand cross-links (51, 52); and two oxidizing agents, potassium bromate and menadione (53, 54). Somewhat surprisingly, most of the DNA-damaging agents did not result in any significant change in the extent of polι ubiquitination even several hours after the initial treatment (Fig. 5, *A–D*). In contrast, in cells treated with 30 μm menadione for 1 h (time 0), we observed an increase of polι with much slower mobility that is consistent with polyubiquitination of polι (Fig. 5*E*). Furthermore, the intracellular levels of polι decreased significantly 3–5 h after treatment, suggesting that the posttranslationally modified polι protein is targeted for degradation.

##### Polι Ubiquitination in Response to Treatment with Various Naphthoquinones and Inhibitors of Mitochondrial Function

**FIGURE 6.**
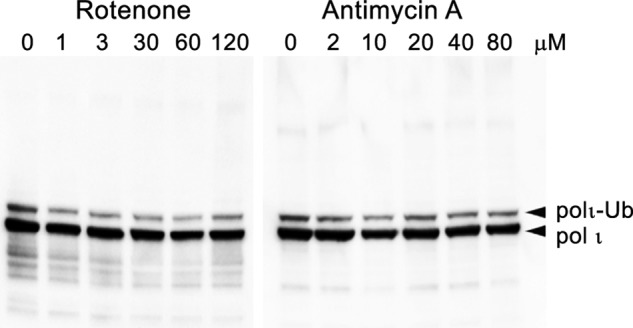
**Effect of rotenone or antimycin A on polι ubiquitination in HEK293T cells.** Cells were treated with the indicated amount of either rotenone or antimycin A for 1 h prior to harvesting. Polι was visualized in Western blots using monoclonal antibodies to the N-terminal FLAG epitope. The major band is unmodified polι followed by monoubiquitinated polι. Under these conditions, there was no significant induction of polyubiquitinated polι. *Ub*, ubiquitin.

Our observation that potassium bromate did not elicit the same polyubiquitination of polι as menadione suggests that polyubiquitination of polι is unlikely to occur as a result of oxidative damage *per se* but occurs in specific response to menadione treatment. Although both agents cause oxidative DNA damage, they do so by different mechanisms. For example, potassium bromate induces glutathione-mediated oxidative base damage (53), whereas menadione does so by inducing mitochondrial dysfunction, leading to an increase in reactive oxygen species (54). However, we observed no significant increase in polι polyubiquitination after inhibition of the mitochondrial respiratory chain complex I with rotenone (55) or antimycin A, which inhibits cytochrome *c* reductase and the production of ATP (56) (Fig. 6), indicating that simple mitochondrial dysfunction is not the root cause for polι polyubiquitination.

**FIGURE 7.**
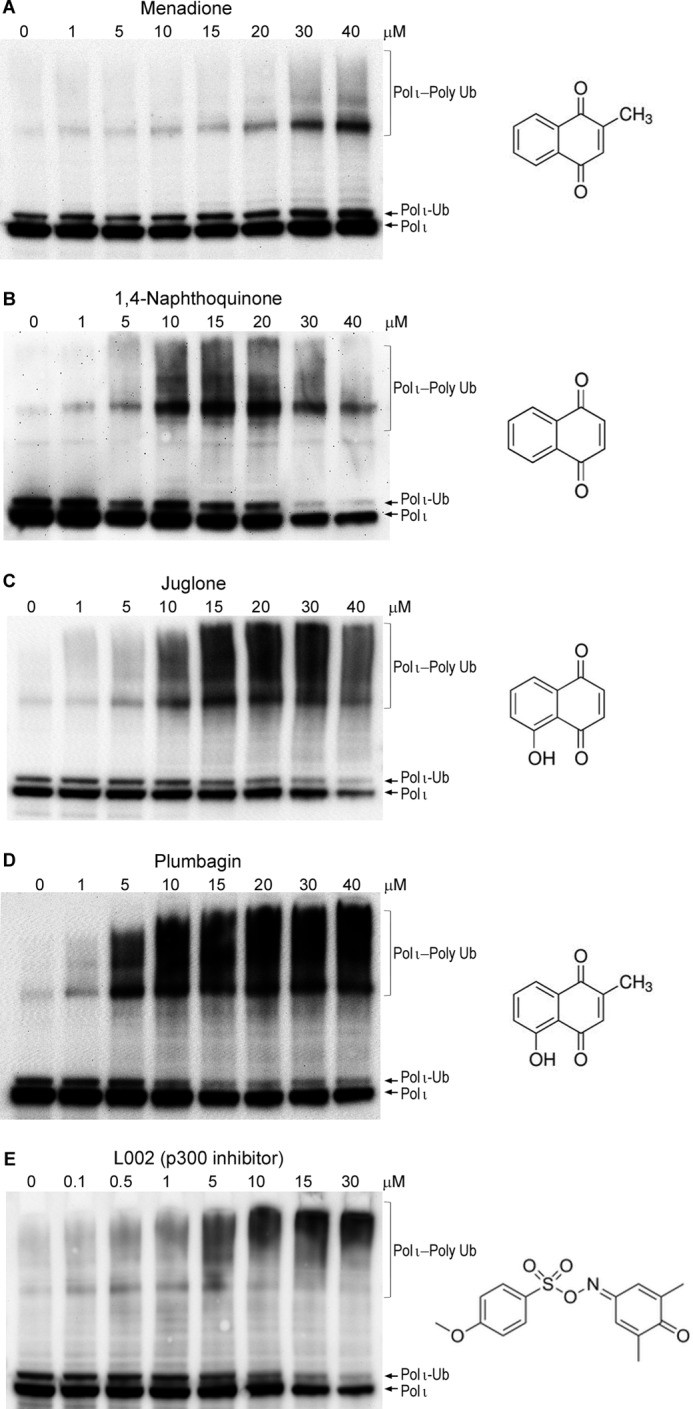
**Effect of various naphthoquinones and a lysine acetyltransferase inhibitor, L002, on the extent of polι polyubiquitination in HEK293T cells.** Cells were treated for 1 h with the indicated concentration of each compound. *A*, menadione; *B*, 1,4-naphthoquinone; *C*, juglone; *D*, plumbagin; *E*, L002. Polι was visualized in Western blots using monoclonal antibodies to the N-terminal FLAG epitope. All compounds lead to an increase in polyubiquitinated forms of polι with the most dramatic effects observed with the naturally occurring naphthoquinone plumbagin. The chemical structures of each compound are shown on the *right-hand side* of each panel. *Ub*, ubiquitin.

**FIGURE 8.**
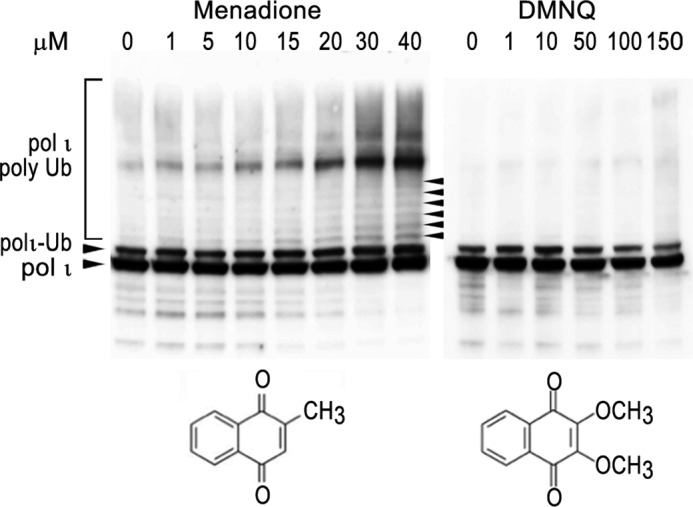
**Comparison of the effect of menadione treatment with DMNQ treatment on polι ubiquitination in HEK293T cells.** Cells were treated with the indicated amount of either menadione or DMNQ for 1 h prior to harvesting. Polι was visualized in Western blots using monoclonal antibodies to the N-terminal FLAG epitope. The panel depicting polι ubiquitination after menadione treatment is a slightly darker exposure of Fig. 7*A* to highlight the laddering of the ubiquitinated forms of polι (as noted by *arrowheads*). Under these conditions, there was no significant induction of polyubiquitinated polι after treatment with DMNQ. *Ub*, ubiquitin.

In contrast, we discovered that polι polyubiquitination occurs after exposure to naphthoquinones that are structurally related to menadione (Fig. 7), including 1,4-naphthoquinone, juglone, and plumbagin. Similar to menadione, all three compounds stimulated polι polyubiquitination in a concentration-dependent manner (Fig. 7). A particularly strong effect was observed after exposure to low concentrations of juglone and plumbagin (Fig. 7, *C* and *D*). However, another naphthoquinone, DMNQ, that also causes significant oxidative DNA damage (57) did not induce polι polyubiquitination (Fig. 8). Although the UV irradiation-induced ubiquitination/deubiquitination of polη and polι seem to be differentially regulated (*c.f.* Ref. 12 and Fig. 5*A*), pol η also appears to undergo polyubiquitination in response to menadione and plumbagin treatment.^3^ Whether this occurs as a result of a common pathway controlling the polyubiquitination of both polymerases in response to naphthoquinones remains to be determined.

##### Polι Ubiquitination in Response to Treatment with an Inhibitor of KAT3B/p300

It is apparent that the effects of menadione, juglone, 1,4-naphthoquinone, and plumbagin cannot be simply attributed to oxidative DNA damage or mitochondrial dysfunction. However, naphthoquinones are also known to inhibit the activity of the lysine acetyltransferase KAT3B/p300 (58, 59). We therefore considered the possibility that the inhibition of ubiquitin acetylation may promote the polyubiquitination of polι. To test this hypothesis, HEK293T cells were treated with the KAT3B/p300 inhibitor L002. Indeed, similar to the effects of naphthoquinones, L002 resulted in the polyubiquitination of polι (Fig. 7*E*).

##### Mass Spectrometry Analysis of Polyubiquitinated Forms of Polι

To further explore the nature of polyubiquitinated forms of polι appearing after treatment with naphthoquinones, we performed mass spectrometry analysis on the purified ubiquitin-conjugated polι. To do so, we used anti-FLAG M2 beads (Sigma) to pull down N-terminal FLAG-tagged polι expressed in HEK293T cells that had been treated for 1 h with 30 μm menadione (Fig. 7*A*) followed by tryptic digestion and LC-MS/MS analysis. This analysis was repeated twice with independently prepared extracts, and extracts from non-treated cells were used as controls. In control experiments, we predominantly observed monoubiquitinated forms of polι. In these extracts, we identified seven ubiquitinated lysines (Lys^87^, Lys^271^, Lys^283^, Lys^309^, Lys^486^, Lys^488^, and Lys^508^). All of them except Lys^508^ were known as potential ubiquitination sites from previous approaches. Interestingly, in extracts prepared from menadione-treated cells where intensive polyubiquitination of polι was observed, we identified four ubiquitinated lysines (Lys^271^, Lys^309^, Lys^320^, and Lys^488^) located in the N-terminal and central parts of polι. Three of them, Lys^271^, Lys^309^, and Lys^488^, were identified in both independent experiments. Because all four residues were previously indicated in our earlier experiments or in proteome-wide experiments (40, 42, 43, 46, 47) as potential ubiquitination sites in untreated cells, we conclude that menadione treatment most probably causes polyubiquitination of polι at lysine residues that are already monoubiquitinated rather than *de novo* at novel lysines.

**FIGURE 9.**
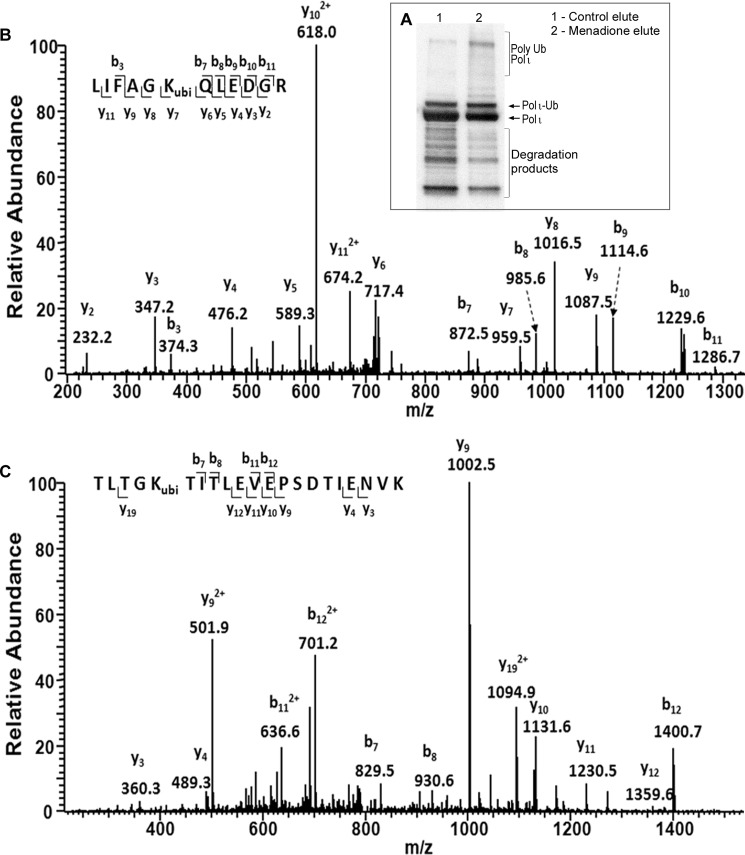
**Mass spectrometry analysis of ubiquitinated polι recovered from menadione-treated HEK293T cells.** *A*, Western blot of purified proteins recovered from untreated and menadione-treated cells. Note that the menadione-treated cell extract contains significantly more polyubiquitinated forms of polι than the untreated cell extract. Both extracts were subjected to mass spectrometry analysis. *B*, the MS/MS of the [M + 2H]^2+^ ion of the peptide ^43^LIFAGKubiQLEDGR^54^ (ubi indicates ubiquitination) from menadione-treated polι samples showing the Lys^48^ linkage of ubiquitin. *C*, the MS/MS of the [M + 2H]^2+^ ion of the peptide ^7^TLTGKubiTITLEVEPSDTIENVK^27^ from menadione-treated polι samples showing the Lys^11^ linkage of ubiquitin. *ubi*, ubiquitination; *Ub*, ubiquitin.

In polyubiquitin chains, ubiquitins are linked to each other via an isopeptide bond between the C-terminal glycine of one ubiquitin and one of the lysine residues of the next ubiquitin. Ubiquitin contains seven lysine residues (Lys^6^, Lys^11^, Lys^27^, Lys^29^, Lys^33^, Lys^48^, and Lys^63^), and all of them can become ubiquitinated to establish polyubiquitin chains of different shape and biological function (for a review, see Ref. 36). Mass spectrometry analysis of polyubiquitinated polι revealed that the polyubiquitin chains formed in response to menadione are formed via Lys^11^ and Lys^48^ linkages (Fig. 9, *B* and *C*). Aside from these two peptides carrying a diglycine remnant, we also observed unmodified tryptic peptides derived from ubiquitin, including TLSDYNIQK (amino acid residues 55–63) and TITLEVEPSDTIENVK (amino acids residues 12–27). Moreover, we observed Lys^11^ and Lys^48^ linkages in polyubiquitinated polι obtained from plumbagin-treated cells.^3^ Presumably, these linkages are formed in response to a common signal induced by exposure to naphthoquinones.

**FIGURE 10.**
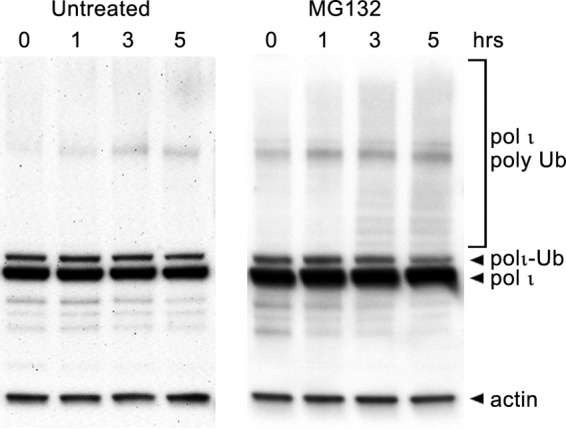
**Effect of the proteasomal inhibitor MG132 on polι ubiquitination in HEK293T cells.** Cells were either untreated or exposed to the proteasomal inhibitor MG132 for the times indicated. Polι was visualized in Western blots using monoclonal antibodies to the N-terminal FLAG epitope. In both cases, the major band is unmodified polι followed by monoubiquitinated polι. We note that the intensity of polyubiquitinated polι increases over time in the MG132-treated cells compared with untreated cells, suggesting that the basal level of polyubiquitinated polι is normally kept to a minimum by 26S proteasomal degradation. *Ub*, ubiquitin.

Lys^48^-linked polyubiquitin chains represent one of the most known and abundant ubiquitin linkages in the cell and target marked proteins to degradation by the 26S proteasome (60, 61). The cellular role of the Lys^11^ linkage is less known; however, the function of homogenous Lys^11^-linked polyubiquitin chains is also implicated in proteasomal degradation (62, 63). Consistent with the notion that polyubiquitinated polι is subject to proteasomal degradation, we observed an increase in the background levels of polyubiquitinated polι in undamaged cells in the presence of the proteasome inhibitor MG132 (Fig. 10).

## Discussion

Ubiquitination is an important factor allowing for quick, controlled, and reversible modification of the fate, cellular abundance, function, and localization of a protein and the promotion of protein-protein interactions. Several proteins utilized in TLS are known to be ubiquitinated *in vivo*, and the protein modification is used to adjust the specificity of TLS mechanisms in a variety of ways (for a review, see Ref. 6).

It has been a decade since the discovery that human polι can be monoubiquitinated *in vivo* (11), but the consequences of the modification remains enigmatic. Our previous studies, which show the dependence of a polι-polη interaction on the ubiquitination of either protein (13), provided some early insights into a possible role of polι modification.

In the present study, we have identified a number of lysine residues in polι that can be covalently linked to ubiquitin. Unlike polη, which is ubiquitinated at one primary site and a handful of secondary sites (6, 12), we discovered that polι is ubiquitinated at more than 27 unique sites (Fig. 4). Two-thirds of the identified sites were detected in multiple autonomous studies using different experimental strategies (*e.g.* ectopic expression of N- and C-terminal FLAG-tagged polι *versus* native untagged chromosomally expressed polι and different methods of identification). Although we cannot exclude the possibility that polι is ubiquitinated at random sites, we believe that the detection of specific ubiquitination sites in multiple independent studies increases the probability that those sites are likely to play key roles in regulating the cellular activities of polι.

When assayed by SDS-PAGE, the predominant form of polι is a single monoubiquitinated species rather than multiply monoubiquitinated forms of polι. We note that under certain conditions, such as when cells are exposed to menadione, we did observe a “laddering” of FLAG-tagged polι, which is indicative of multiple monoubiquitination events (Fig. 7), but we cannot distinguish between the possibility of multiple monoubiquitinations of polι or a single monoubiquitination event that is subsequently converted into a polyubiquitin chain. Our observations therefore indicate that once polι is monoubiquitinated at one particular site it subsequently precludes monoubiquitination at additional sites in polι. Clearly, this is an area of research that needs to be studied in detail and will be the subject of future studies.

**FIGURE 11.**
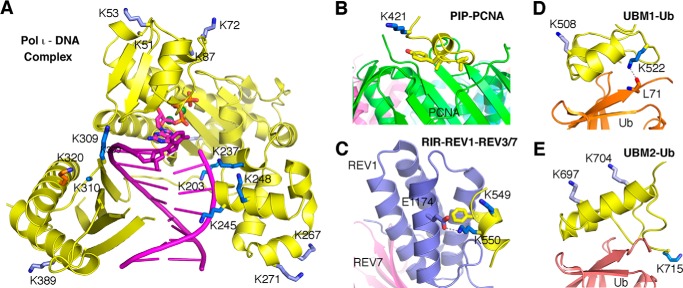
**Diagram of ubiquitinated lysine residues in the three-dimensional structures of human polι**. *A*, the catalytic domain of human polι (amino acids 1–420) is shown in a ternary complex with DNA (*purple tube* and *ladder*) and an incoming dGTP (Protein Data Bank code 3GV8) (29). The polι regions are always shown as a *yellow* schematic with the lysine located near the functional interface highlighted as a *dark blue stick*. The lysines that are distal from a functional surface are shown in *light blue*. Lys^320^, whose ubiquitination may destabilize the protein structure, is shown in *orange*. Some lysine side chains are disordered and are thus modeled as alanine (Lys^51^, Lys^83^, Lys^283^, and Lys^310^). *B*, the PCNA-interacting peptide motif (*PIP*) region of human polι. Lys^421^ is near the interface with the *green* subunit of the trimeric PCNA (the other two PCNA subunits are shown in *pink* and *cyan* behind the *green* subunit) according to the crystal structure (Protein Data Bank code 2ZVM) (32). *C*, the Rev1-interacting region (*RIR*) of human polι is modeled after the crystal structures of human and mouse polκ in a complex with REV1/3/7 (Protein Data Bank codes 4GK5 and 4FJO, respectively). Two lysine residues of the Rev1-interacting region (Lys^549^-Lys^550^) are conserved, but only one (Lys^550^) is near the interface with REV3 and forms a salt bridge with a conserved glutamate of REV1 (Glu^1174^ in human REV1). *D*, the UBM1 of human polι is modeled after the NMR structure of mouse UBM1 in a complex with ubiquitin (*Ub*) (Protein Data Bank code 2KWV) (69). Lys^522^ is hydrogen-bonded with the main chain carbonyl oxygen of Leu^71^ in ubiquitin. *E*, UBM2 of human polι in complex with ubiquitin is shown according to the NMR structure (Protein Data Bank code 2L0F) (70). Lys^715^, the C-terminal residue of polι, is near the interface with ubiquitin, whereas Lys^697^ and Lys^704^ are distal from ubiquitin.

No single Lys → Ala or Lys → Arg substitution completely blocked monoubiquitination of polι. However a Lys → Arg substitution at Lys^715^, which is located at the very C terminus of polι and which has been shown to be ubiquitinated in two independent proteome-wide approaches (43, 49), gave the greatest reduction in monoubiquitination (Fig. 3). We predict that the structure of ubiquitin covalently linked to Lys^715^ will position the ubiquitin moiety for a productive interaction with the UBM2 of polι (Fig. 11*E*). Similarly, ubiquitination at Lys^522^ may also facilitate an interaction between ubiquitin and UBM1 of polι (Fig. 11*D*). We hypothesize that such interactions may, in turn, help promote an interaction between polι and polη (13).

In contrast, monoubiquitination of other lysine residues may have a detrimental effect on the cellular functions of polι. For example, many sites are located in the catalytic domain of the polymerase and may alter both DNA binding properties and polymerase activity of polι (Figs. 4 and 11*A* and Table 2). Ubiquitination sites were also identified in both the PCNA-interacting peptide motif box and the Rev1-interacting region motif, and it seems highly unlikely that polι would be able to physically interact with either PCNA or Rev1 if these sites are ubiquitinated (Fig. 11, *B* and *C*).

**TABLE 2:** **Location of ubiquitination sites in polι and their structural implications** See Fig. 11. ssDNA, single-stranded DNA.

**A: polymerase domain**
Lys^51^: located in the finger domain, pointing toward the outside of the protein
Lys^53^: located in the finger domain, pointing toward the outside of the protein
Lys^72^: located in the finger domain, pointing toward the outside of the protein
Lys^87^: located in the finger domain ∼20 Å from the incoming nucleotide; may have some effect on catalysis
Lys^138^: on the rear side of the protein when looking at the active site of the polymerase
Lys^203^: likely to affect DNA binding and overall structure of the polymerase
Lys^237^: likely to affect DNA binding
Lys^245^: likely to affect DNA binding
Lys^248^: likely to affect DNA binding
Lys^267^: on the rear side of the protein when looking at the active site of the polymerase
Lys^271^: ∼15 Å from the upstream DNA duplex; may have some effect on catalysis
Lys^283^: on the rear side of the protein when looking at the active site of the polymerase
Lys^309^: may be involved in DNA binding as it is ∼8 Å from the downstream ssDNA
Lys^310^: on the same face as DNA binding but distal from DNA
Lys^320^: near Asp^306^ and Glu^323^ for the structure stability and near the finger domain
Lys^389^: on the same face of DNA binding but distal from DNA
**B. PCNA interaction motif**
Lys^421^: near the PCNA interface
**C. UBM1**
Lys^508^: conserved in mouse (Lys^506^) and pointing away from the ubiquitin interface
Lys^522^: conserved in mouse (Lys^520^) and forming an H-bond with ubiquitin
Lys^526^: not conserved in mouse UBM1; Asn^524^ of mouse polι points away from the ubiquitin interface
Lys^530^: not in the mouse UBM1 structure
**D. Rev1-interacting region**
Lys^549^: pointing away from the interface with Rev1
Lys^550^: close to the surface of Rev1
**E. UBM2**
Lys^697^: pointing away from ubiquitin
Lys^704^: pointing away from ubiquitin
Lys^715^: C-terminal residue; not in UBM2 *per se*, but conjugation at this residue would likely position ubiquitin for a non-covalent interaction with UBM2

Unlike polη, which is deubiquitinated upon UV irradiation (12), the level of polι monoubiquitination remained constant after exposure to a variety of DNA-damaging agents, including UV light, ethyl methanesulfonate, mitomycin C, or the oxidative DNA damage inducers potassium bromate (Fig. 5), rotenone and antimycin A (Fig. 6), and DMNQ (Fig. 8). In dramatic contrast, menadione and several structurally related naphthoquinones resulted in the rapid polyubiquitination of polι. Mass spectrometry of polyubiquitinated polι purified from menadione- and plumbagin-treated cells indicated that the polyubiquitin chains were formed through Lys^11^ and Lys^48^ linkages. Conjugation of ubiquitin via Lys^48^ linkage is well known to serve as a signal for proteasomal degradation (60, 61). Indeed, the disappearance of polι 3–5 h after exposure to menadione (Fig. 5*E*) is consistent with its degradation.

We initially considered that the signal triggering polyubiquitination of polι might be oxidative DNA damage, but this was rapidly excluded when we failed to observe polyubiquitination in response to potassium bromate (Fig. 5*C*), DMNQ (Fig. 8), rotenone, or antimycin A (Fig. 6). However, in addition to the induction of reactive oxygen species, the naphthoquinones are also known to exert a wide range of cellular effects leading to stress signaling, antiangiogenesis, and thiolate arylation of proteins and amines (64–66). One property of interest is their ability to inhibit the lysine acetyltransferase (KAT) p300 (58, 59). All of the naphthoquinones (1,4-naphthoquinone, menadione, juglone, and plumbagin) that induced polι polyubiquitination have previously been reported to inhibit the KAT activity of p300 (59). It is unknown whether DMNQ (which did not cause polι polyubiquitination) can inhibit the KAT activity of p300. However, because DMNQ lacks the critical hydroxyl group at the fifth position of the benzene ring that is required for inhibition of KAT activity (59) and the second and third positions that are normally subject to nucleophilic attack are occupied by methoxy groups (Fig. 8), we assume that it probably does not inhibit KAT activity.

It is now well established that acetylation is a key regulator of diverse biological processes from metabolism to signaling and immunity (67). Indeed, like many proteins, ubiquitin is subject to acetylation (49, 68). Interestingly, both Lys^11^ and Lys^48^ are moderately sensitive to acetylation (49, 68). Thus, we hypothesize that if Lys^11^ and Lys^48^ of ubiquitin are acetylated it would preclude the formation of polyubiquitin chains via these linkages. Our observation that the p300 inhibitor L002 also induces polyubiquitination of polι strongly suggests that there is a competition between ubiquitination and acetylation at overlapping lysine residues in polι. We believe that such competition constitutes a novel mechanism to regulate the stability of polι that warrants further investigation.

## Author Contributions

J. M. constructed all of the pJRM expression plasmids shown in Table 1 as well as designed, performed, and analyzed the experiments shown in Figs. 2, 3, and 4 and wrote the paper. M. P. M. performed and analyzed the experiments shown in Figs. 1, 5, 6, 7, 8, and 10. E. G. F. prepared purified N-terminal FLAG-tagged polι for mass spectrometry analysis shown in Fig. 4. X. D. and Y. W. designed, performed, and analyzed the mass spectrometry experiments shown in Figs. 4 and 9. W. Y. analyzed the structural ramifications of polι monoubiquitination shown in Fig. 11 and Table 2. R. W. conceived and coordinated the study and wrote the paper. All authors reviewed the results and approved the final version of the manuscript.

## Footnotes

3 J. McIntyre, M. P. McLenigan, E. G. Frank, X. Dai, W. Yang, Y. Wang, and R. Woodgate, unpublished observations.

## Footnotes

2 The abbreviations used are:

TLS: translesion DNA synthesis
pol: DNA polymerase
PCNA: proliferating cell nuclear antigen
UBM: ubiquitin binding motif
DMNQ: 2,3-dimethoxy-1,4-naphthoquinone
KAT: lysine acetyltransferase.
